# Co-evolution of machine learning and digital technologies to improve monitoring of Parkinson’s disease motor symptoms

**DOI:** 10.1038/s41746-022-00568-y

**Published:** 2022-03-18

**Authors:** Anirudha S. Chandrabhatla, I. Jonathan Pomeraniec, Alexander Ksendzovsky

**Affiliations:** 1 School of Medicine, University of Virginia Health Sciences Center, Charlottesville, VA 22903 USA; 2 Surgical Neurology Branch, National Institutes of Neurological Disorders and Stroke, National Institutes of Health, Bethesda, MD 20892 USA; 3 Department of Neurosurgery, University of Virginia Health Sciences Center, Charlottesville, VA 22903 USA; 4 Department of Neurosurgery, University of Maryland Medical System, Baltimore, MD 21201 USA

**Keywords:** Translational research, Learning algorithms, Technology

## Abstract

Parkinson’s disease (PD) is a neurodegenerative disorder characterized by motor impairments such as tremor, bradykinesia, dyskinesia, and gait abnormalities. Current protocols assess PD symptoms during clinic visits and can be subjective. Patient diaries can help clinicians evaluate at-home symptoms, but can be incomplete or inaccurate. Therefore, researchers have developed in-home automated methods to monitor PD symptoms to enable data-driven PD diagnosis and management. We queried the US National Library of Medicine PubMed database to analyze the progression of the technologies and computational/machine learning methods used to monitor common motor PD symptoms. A sub-set of roughly 12,000 papers was reviewed that best characterized the machine learning and technology timelines that manifested from reviewing the literature. The technology used to monitor PD motor symptoms has advanced significantly in the past five decades. Early monitoring began with in-lab devices such as needle-based EMG, transitioned to in-lab accelerometers/gyroscopes, then to wearable accelerometers/gyroscopes, and finally to phone and mobile & web application-based in-home monitoring. Significant progress has also been made with respect to the use of machine learning algorithms to classify PD patients. Using data from different devices (e.g., video cameras, phone-based accelerometers), researchers have designed neural network and non-neural network-based machine learning algorithms to categorize PD patients across tremor, gait, bradykinesia, and dyskinesia. The five-decade co-evolution of technology and computational techniques used to monitor PD motor symptoms has driven significant progress that is enabling the shift from in-lab/clinic to in-home monitoring of PD symptoms.

## Introduction

Parkinson’s disease (PD) is a complex neurodegenerative disorder commonly characterized by motor impairments such as tremor, bradykinesia, dyskinesia, and gait abnormalities^1^. Proper assessment of PD motor impairments is vital for clinical management of the disease^2,3^. Appropriate timing of dopaminergic medications^4^ to avoid sudden increases in symptom severity^5^ and selection for interventions such as deep brain stimulation^6^ both require precise understandings of symptom fluctuations in patients with PD. In addition, objective characterization of non-motor manifestations of PD such as sleep disorders, gastrointestinal symptoms, and psychiatric symptoms are needed to understand long-term disease progression^3^.

Characterization of motor and non-motor PD symptoms traditionally relied on the Unified Parkinson’s Disease Rating Scale (UPDRS), a PD severity rating system with four parts related to (I) Mentation, Behavior and Mood, (II) Activities of Daily Living, (III) Motor, and (IV) Complications^7^. The UPDRS was eventually updated by the Movement Disorder Society (MDS), creating the MDS-UPDRS, in an attempt to reduce subjectivity in the scale^8^. Clinicians also use other rating systems such as the WHIGET Tremor Rating Scale for action tremor^9^ and the modified bradykinesia rating scale (MRBS) for bradykinesia^10^. However, these rating systems suffer from two main flaws. First, they lack granularity during disease or medication cycles, as they only provide a snapshot view of a patient’s symptoms as seen during in-clinic visits. In addition, when assessing PD symptoms outside of the clinic, physicians must rely on patient diaries or recall, which can be inaccurate^2^. Second, these rating systems are inherently subjective, leading to high inter- and intra-rater variability^3^.

Addressing these flaws is vital to ensure proper diagnosis and management of patients with PD. To that end, considerable efforts have been made to develop objective, at-home, and automated methods to monitor the main motor symptoms characteristic of PD. Leveraging motion sensor and, in some instances, video-based technologies can first enable physicians to take data-driven approaches to PD diagnoses. Adding at-home patient monitoring through smart devices (e.g., smartphones, watches) could then enable physicians to adjust treatment plans based on patient activity data. The end goal of these technologies is to achieve continuous, at-home monitoring, which will require continued research using data from at-home, continuous studies, rather than applying laboratory data to develop at-home solutions. This review aims to summarize the co-evolution of the technologies and computational methods used to assess and monitor common motor symptoms of PD such as tremor, gait abnormalities, bradykinesia, and dyskinesia.

### Review of literature

#### Technology

**Fig. 1:**
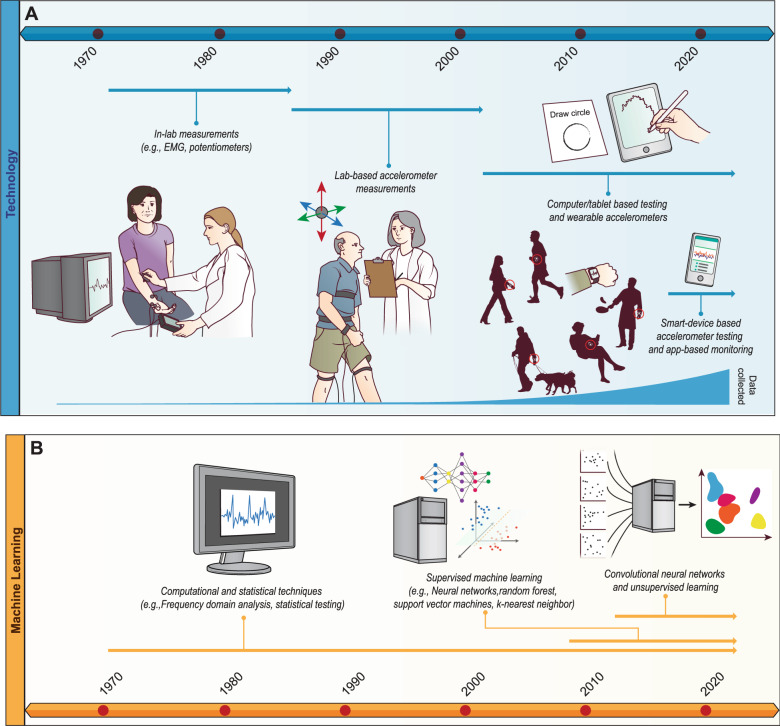
A 50 + year timeline illustrating the progression of technology used to assess and monitor symptoms in patients with PD and illustrating the progression of computational and machine learning techniques used to assess and monitor symptoms in patients with PD. **A** In the 1970s, the main technologies used were lab-based, such as EMG and potentiometer measurements. Adoption of lab-based accelerometers began in the late 1980s and continued until the early 2000s when smaller devices such as tablets and wearable accelerometers started being leveraged. Since the late 2010s, smart devices and apps on those devices were the primary technologies used for symptom monitoring. Over time, the evolution of technology has enabled greater and more continuous data collection. **B** Since the 1970s, computational and statistical techniques such as frequency domain analyses of accelerometer data have enabled researchers and clinicians to quantify symptom severity in patients with PD. Improvements in technologies used to monitor symptoms have enabled increased data collection, allowing for the growth in adoption of machine learning techniques. Supervised techniques were applied first to analyze symptom data, followed by unsupervised techniques.

The technology used to diagnose and monitor PD has evolved significantly over time (Fig. 1A). Most notably, this technology has progressed from laboratory to at-home/everyday settings, enabling more robust data collection related to PD symptoms. This section describes this progression, along with the purpose and advantages of different technologies.

Laboratory-based technologies to assess PD symptoms had two main purposes: (1) develop methodologies to diagnose PD/categorize severity (e.g., distinguish PD from similar neurological conditions) and (2) set the foundation for smaller, more portable, and more user-friendly technologies that could assist in PD diagnosis and monitoring in the future (Table 1).

**Table 1:** Progression of technology used to monitor and assess PD symptoms in laboratory/clinic settings.

Authors, Years	Device	Primary Symptom(s) Measured
Andrews et al.^13^	Surface EMG	Freezing of gait
Milner-Brown et al.^13^	EMG	Bradykinesia
Bathien et al.^11^	EMG	Resting tremor and dyskinesia
Hacisalihzade et al.^14^	In-lab potentiometer-based motion tracker	Bradykinesia while tracking moving target
van Hilten et al.^44^	Accelerometer on non-dominant wrist	Continuous monitoring of tremor and dyskinesia
Weller et al.^16^	Infrared-based shoe sensor	Straight-line gait
Beuter et al.^15^	In-lab laser-based system	Resting and action tremor
Deuschl et al.^141^	Monoaxial accelerometer	Resting tremor
Dunnewold et al.^24^	Tri-axial accelerometer	Bradykinesia
Someren et al.^142^	Uniaxial accelerometer	Tremor
Dunnewold et al.^22^	Uniaxial accelerometer	Bradykinesia, hypokinesia,
Spyers-Ashby et al.^19^	Tri-axial accelerometer	Postural tremor
Giovannoni et al.^38^	Computer keyboard to administer the *BRAIN TEST*	Bradykinesia while alternately striking computer keys for a period of 60 seconds.
Rajaram et al.^20^	Tri-axial electromagnetic sensors	Resting, postural, and intention tremor. Also included distraction and mental stress conditions.
Manson et al.^35^	Tri-axial accelerometer on shoulder	Dyskinesia in multiple conditions (e.g., sitting, writing)
O’Suilleabhain. et al.^17^	Electromagnetic motion tracking system	Quantitative tremor assessment in multiple conditions (e.g., arms horizontal and straight ahead, shoulders abducted to 90°)
Hoff et al.^23^	Bi-axial accelerometers	Dyskinesia during rest, talking, stress, and four activities of daily life (ADL
Burne et al.^45^	Tri-axial accelerometer and surface EMG	Resting and postural tremor
Hoff et al.^21^,	Uniaxial accelerometers	“On” and “off” tremor states
Sekine et al.^143^	Tri-axial accelerometer and photoelectric sensor	Gait
Salarian et al.^25^	Tri-axial gyroscope	Bradykinesia and tremor while performing activities of daily life (e.g., brushing hair and teeth, putting on and taking off a jacket and shoes)
Allen et al.^39^	Computer with videogame joystick and steering wheel	Bradykinesia while using videogame joystick and steering wheel
Rao et al.^43^	Video	Dyskinesia (face and neck) during speech task
Giansanti et al.^144^	Force sensor/step counter	Gait
Salarian et al.^29^	Tri-axial accelerometer and gyroscope	Straight-line gait with turning
Mancini et al.^145^	Tri-axial accelerometers and gyroscopes. Force plate	Bradykinesia
Bachlin et al.^41^	Accelerometer and headphones for audio cues	Freezing of gait
Cole et al.^46^	Tri-axial accelerometer and surface EMG	Scripted (e.g., tooth-brushing) and unscripted action tremor
Espay et al.^40^	4 m electronic walkway. VR goggles and earphones	Straight-line gait with or without feedback from goggles and earphones
Papapetropoulos et al.^48^	Tremor pen with bi-axial accelerometer, touch recording plate, reaction time handle, and force plate	Postural and action tremor (with distraction conditions), reaction time, and postural stability
Mancini et al.^146^	Tri-axial accelerometer and gyroscope. Force plate	Gait (via postural sway)
Heldman et al.^27^	KinetiSense motion sensor on heel	Bradykinesia
Tsipouras et al.^47^	Tri-axial accelerometers and gyroscopes	Action tremor in scripted conditions (e.g., rising from bed and sitting on chair)
Mera et al.^26^	Tri-axial accelerometer and gyroscope	Bradykinesia and tremor in multiple conditions (e.g., rest, repetitive finger-tapping)
Moore et al.^30^	7 inertial measurement units	Freezing of gait from timed up-and-go tasks
Tripoliti et al.^33^	6 accelerometers and gyroscopes	Freezing of gait during simulated activities of daily life
Morris et al.^147^	Animations generated from inertial sensors	Freezing of gait
Zach et al.^34^	Tri-axial linear waist-mounted accelerometer	Freezing of gait during walking tasks
Ginis et al.^148^	Inertial measurement units and smartphone app	Gait
Phan et al.^28^	Tri-axial accelerometer, gyroscope, and compass	Axial bradykinesia in multiple conditions (e.g., pouring water from a jug into 9 cups)
Pulliam et al.^36^	*Kinesia* motion sensor (tri-axial accelerometer and gyroscope) on each wrist and ankle.	Motor fluctuations (“ON” vs “OFF” state) during simulated activities of daily life
Rodriguez-Molinero et al.^37^	Waist-worn accelerometer, gyroscope, magnetometer	Dyskinesia while performing activities of daily life (e.g., brushing teeth, drying a glass)
Reches et al.^32^	*Opal* sensors (tri-axial accelerometer, gyroscope, and magnetometer)	Freezing of gait during walking tasks
Lee et al.^42^	*Google Glass*	Freezing of gait during walking tasks
Mancini et al.^31^	*Opal* sensors (tri-axial accelerometer, gyroscope, and magnetometer)	Freezing of gait during walking tasks and during activities of daily life

All data were collected in controlled environments (e.g., laboratories, hospitals).

Laboratory-based electromyography (EMG) techniques were among the first technologies used to assess PD. More specifically, the data collected using these techniques was primarily meant to help distinguish/diagnose PD from similar conditions or quantify disease progression. In 1984, Bathien et al. quantified tremor of the head, hands, and lower extremities with EMG^11^. The group found that analyzing phase-shifts between bursts of EMG activity in agonist-antagonist muscles enabled categorization between the tremor seen in PD and that of tardive dyskinesia, creating one of the first quantitative methodologies for distinguishing PD from other conditions. In-lab EMG was also leveraged to quantify and monitor gait abnormalities in patients with PD. EMG data helped distinguish between normal and “Parkinsonian” gait and quantify response to therapy over time^12^. Similar studies were conducted to assess other symptoms of PD. In 1979, Milner-Brown et al. reported that needle-based hand EMG detected abnormal motor unit properties during muscle contraction that could be used to track progression of bradykinesia^13^. Of note, these EMG-based techniques were not meant for making initial PD diagnoses, but were rather used for tracking progression of already established disease.

Starting in the latter half of the 1980s, researchers began moving past EMG and towards less invasive methods. The technologies developed during this era also attempted to diagnose PD in addition to monitoring/quantifying symptoms. The initial techniques developed varied widely. Some groups tested potentiometer-based systems that could monitor multiple symptoms at once^14^, allowing for “one stop” assessments of, for example, how patients were responding to pharmacologic therapy. Laser-based technologies were also popular. Beuter et al. developed a laser system that could measure hand movements to distinguish between healthy controls and patients with PD^15^, while Weller et al. developed a system to track how gait abnormalities changed in response to various medications^16^. Though these technologies were less invasive and more portable than EMG, their use was often limited to special laboratory environments (e.g., areas with laser-safety equipment) and required significant expertise to operate^17^. Accelerometers and gyroscopes addressed both of these concerns, thereby solidifying them as two of the main technologies that defined the next era of PD monitoring. The use of accelerometers and gyroscopes enabled increased data collection, thereby improving the granularity with which researchers were able to monitor and assess patients with PD. Early use of accelerometers and gyroscopes collected in-lab data in one axis and looked to differentiate between PD and other conditions. Deuschl et al. used a monoaxial accelerometer to demonstrate that time series analysis alone was sufficient to differentiate between PD and essential tremor^18^. The use of tri-axial accelerometers and gyroscopes improved classification accuracy and allowed for more robust in-lab measurements. The tri-axial accelerometers employed by Spyers-Ashby et al. in 1999 lead to greater than 60% classification accuracy between control, essential tremor, multiple sclerosis, and PD^19^. Additionally, Rajaraman et al. demonstrated that using an increased number of tri-axial accelerometers on various parts of the hand, forearm, and arm allowed for quantification of tremor despite altered hand positions and orientation^20^. Seminal studies by the van Hilten group also demonstrated that tri-axial accelerometry was beneficial in identifying and characterizing tremor, bradykinesia, and dyskinesia^21–24^.

The use of wearable accelerometers and gyroscopes extended to quantifying other PD symptoms. Data from tri-axial accelerometers and gyroscopes on various parts of the body (e.g., wrists, index finger, back) allowed for models to estimate UPDRS scores and determine bradykinesia severity under both scripted and unscripted conditions^25–28^. Salarian et al. investigated using tri-axial accelerometers and gyroscopes along with inertial sensors to track postural instability gait difficulty (PIGD sub score of UPDRS III) during gait-assessment turning trials, reporting that patients with PD had significantly longer turning duration and delay before initiating a turn^29^. Similar findings were reported by Moore et al., who showed that freezing-of-gait (FoG) identification based on frequency characteristics of lower extremity motion correlated strongly (interclass correlation >0.7) with clinical assessments by specialists^30^. The use of accelerometers to identify FoG has been reported by many other groups as well^31–34^. Multiple studies investigating dyskinesia severity used tri-axial accelerometers, gyroscopes, and/or magnetometers on various body parts (e.g., shoulder, wrist, ankle, waist) and found strong correlations between the magnitudes of dyskinesia measured by devices to those observed by clinicians^35–37^.

At the same time, in-lab methodologies were being developed specific to quantifying and better understanding certain manifestations of PD. Unique to bradykinesia was the use of computer game-based technologies. In 1999, Giovannoni et al. introduced the *BRAIN TEST* as a computer-based way to monitor the progression of bradykinesia in PD. By requiring participants to use their index fingers to alternately strike the “S” and “;” keys on a standard computer keyboard, the *BRAIN TEST* provided a rapid and objective measurement of upper-limb motor function^38^. Allen et al. built upon Giovannoni’s work and developed a joystick and toy steering wheel-based computer test that was able to discriminate pathologic bradykinesia of varying severity^39^. Espay et al. studied the effect of virtual reality (VR) and audio-based gait feedback in identifying and correcting gait abnormalities in PD patients as they walked on an in-lab four meter *GAITRite* electronic walkway. Overall, nearly 70% of patients improved by at least 20% in either walking velocity, stride length, or both^40^. Bachlin et al. developed a similar correction-focused platform that detected FoG in patients with PD and provided audio cues to resume walking. The system detected FoG events in real-time with a sensitivity of 73% and specificity of 82%^41^. Visually cued FoG correction platforms have been developed using technologies such as *Google Glass*^42^. Finally, Rao et al. reported a video-based facial tracking algorithm that assessed severity of face and neck dyskinesia during a speech task. The calculated severity scores showed a high correlation to dyskinesia ratings by neurologists^43^.

Leveraging the data and analyses from in-lab studies, researchers began to develop methodologies for not just monitoring, but also diagnosing PD outside of the lab. Initial studies in this area included work by van Hilten et al. in which patients wore small accelerometers over the course of six days and completed quality of life surveys, enabling the first objective measures of dyskinesia^44^. Tremor analyses continued incorporating progressively more wearable accelerometers and enabling accurate classification between PD, essential tremor patients and controls while starting to step outside the boundaries of the lab^45,46^. Tsipouras et al. demonstrated that using multiple, wearable accelerometers and gyroscopes allowed for effective monitoring of patients while performing activities of daily life under real-life, but simulated, conditions^47^. Finally, using accelerometers embedded in a pen along with other sensors (e.g., touch recording plate), Papapetropoulos et al. showed the ability of multiple, small sensors to discriminate types of pathological tremor^48^.

Over the past decade, monitoring of PD symptoms has experienced two thematic changes. First, monitoring has become more remote and accessible due to the ease of use and widespread availability of more wearable accelerometers/gyroscopes and smartphones with those devices built-in. Second, monitoring has become more continuous through the use of web and mobile applications. Together, these changes are making way for more smart technology-mediated assessment of PD, with platforms for diagnosis currently in development (Table 2).

**Table 2:** Progression of technology used to monitor and assess PD symptoms in the home setting.

Authors, Years	Primary study setting	Device	Primary symptom(s) measured
Holmes et al.^134^	Controlled	Professional microphone	Voice recordings
Keijsers et al.^68^	Controlled (home-like)	Tri-axial accelerometers	Fluctuations in Levodopa-induced dyskinesia
Banaszkiewicz et al.^64^	Controlled	Tablet with stylus	Bradykinesia while drawing spirals
Patel et al.^69^	Controlled	Uniaxial accelerometers	Tremor, bradykinesia and dyskinesia severity and fluctuation
Chen et al.^58^	Home	Wearable sensors, web services for live streaming and storage of data, and web-based graphical user interface client	Home monitoring of tremor, bradykinesia, dyskinesia, medication compliance, and other qualitative patient data
Chen et al.^58^	Controlled	In-lab video	Straight-line gait
Kostikis et al.^62^	Controlled	iPhone	Postural tremor
Yang et al.^49^	Controlled	Tri-axial accelerometer	Straight-line gait
Cavanaugh et al.^149^	Home	*StepWatch 3 Step Activity Monitor*	Number of complete gait cycles completed
Cancela et al.^59^	Home	Web interface, tri-axial accelerometers, and belt sensor with accelerometer and gyroscope	Monitoring of tremor, medication (dose, time), meals (type of food, amount, time), and PDQ-39
Daneault et al.^60^	Controlled	Smartphone with accelerometer	Resting, postural, intention, and kinetic tremor
Klucken et al.^50^	Controlled	Tri-axial gyroscopes and accelerometers	Straight-line gait and lower extremity coordination (e.g., heel/toe tapping)
Cancela et al.^59^	Home	Four tri-axial accelerometers. Waist-worn accelerometer and gyroscope.	Gait and bradykinesia
Ferreira et al.^54^	Home	Accelerometers and angular rate sensors, *Wii Balance Board, SENSE-PARK app*	Feasibility of and compliance with continuous sensor-based monitoring
Fisher et al.^55^	Home	Tri-axial accelerometers	Feasibility of and compliance with continuous sensor-based monitoring.
Bank et al.^76^	Controlled	In-lab video	Bradykinesia
Heldman et al.^53^	Home	Wireless motion sensor and touch screen tablet	Resting and postural tremor and bradykinesia
Silva de Lima et al.^65^	Home	“Fox Wearable Companion” app on a smartwatch and smartphone	Continuous monitoring of tremor and qualitative surveys on quality of life
Lakshminarayana et al.^150^	Home	Smartphone with *uMotif* app	Motor: Bradykinesia Other: Sleep, mood, cognition
Rusz et al.^79^	Controlled	Smartphone and professional microphone	Voice recordings
Zhan et al.^72^	Home	Smartphone	Gait, finger tapping, and voice samples
Lo et al.^61^	Home	Smartphone (7 + models were used)	Falls, FoG, postural instability, cognitive impairment, difficulty doing hobbies, need for help at home
Prince et al.^66^	Home	iPhone	Dexterity, gait, phonation, and memory
Isaacson et al.^151^	Home	*Kinesia 360* motion sensor and mobile application	Tremor, bradykinesia and dyskinesia severity and fluctuation
Aich et al.^71^	Controlled	Tri-axial accelerometers	Gait kinematic features
Erb et al.^57^	Controlled (home-like) and Home	Accelerometers, gyroscopes, magnetometers, barometers, EKG, EMG, and/or galvanic skin response	Feasibility of and compliance with continuous sensor-based monitoring Dynamics of tremor, dyskinesia, and bradykinesia over the medication cycle
Evers et al.^56^	Home	Accelerometer, gyroscope, magnetometer, barometer, galvanic skin response, photoplethysmogram, thermometer, and *Android* smartphone	Gait abnormalities via unscripted, real-world collection
Ghoraani et al.^70^	Controlled (home-like)	Tri-axial gyroscopes	Fluctuations between medication ON and OFF states
Lu et al.^74^	Controlled	Video	Straight-line gait
Mahadevan et al.^52^	Controlled	Tri-axial accelerometer, gyroscope, and magnetometer	Identification of resting tremor constancy, bradykinesia MDS-UPDRS scores, and gait abnormalities. Assessment of medication state (“ON” or “OFF”) related changes in tremor
Pfister et al.^73^	Controlled	Microsoft Band 2 (Tri-axial accelerometer and gyroscope along with Bluetooth capabilities)	Fluctuations between “ON”, “OFF”, and “Dyskinetic” states
Sajal et al.^80^	Controlled	Smartphone-based accelerometer	Tremor and voice recordings
Singh et al.^78^	Home	Smartphone	Voice recordings
van Brummelen et al.^63^	Controlled	7 consumer product accelerometers (CPAs) (e.g., iPhone 7, iPod Touch 5) and laboratory-grade accelerometer (Biometrics ACL300)	Postural and action tremor
Dominey et al.^152^	Home	*KinetiGraph* wrist-worn movement recording system.	Motor: tremor, bradykinesia, and dyskinesia. Other: Immobility/somnolence and medication adherence
Gatsios et al.^135^	Home	*Microsoft* Band, a sensor insoles, smartphone with an Android app, and a cloud backend	Motor: Tremor, gait, weight-bearing Other: Heart rate, skin temperature, sleep quality/duration
Daneault et al.^67^	Home	Smartwatch and smartphone with “Fox Wearable Companion” app	Bradykinesia and tremor from continuous home monitoring and in-lab tasks (e.g., finger-to-nose, typing on a keyboard)
Marcante et al.^51^	Controlled (home-like)	Insole sensors consisting of 13 pressure sensors and a tri-axial accelerometer	Gait in scripted scenarios (e.g., rise from bed walk to chair)
Powers et al.^2^	Home	Apple Watch	Fluctuations in resting tremor and dyskinesia. Tremor severity and presence of dyskinesia
Hadley et al.^153^	Home	Smartphone and smartwatch	Tremor, bradykinesia and dyskinesia severity and fluctuation
Sundgren et al.^154^	Home	*KinetiGraph* wrist-worn movement recording system.	Motor: tremor, bradykinesia, and dyskinesia. Other: Immobility/somnolence and medication adherence

Data were either collected in controlled environments and applied to the home setting or were directly collected in home settings.

Wearable sensors are making way for more remote assessment of PD symptoms. Yang et al. found that a single, small tri-axial accelerometer attached to the belt buckle enabled estimation of multiple gait parameters such as cadence, step regularity, stride regularity and step symmetry to be estimated in real-time, allowing for immediate quantification of gait^49^. Klucken et al. also reported the use of a small, heel-clipped device that achieved a classification accuracy of 81% differentiating between PD patients and healthy controls^50^. More recently, a study of insole sensors enabled detection of PD-related FoG episodes with 90% accuracy^51^ and wrist-worn accelerometers achieved “good to strong” agreement with clinical ratings of resting tremor and bradykinesia, in addition to discriminating between treatment-caused changes in motor symptoms^52^. Though some of these studies were conducted in laboratory settings, the collective results indicate that patients could wear similar devices at home, enabling remote mobility assessment. Studies specifically assessing wearable technologies’ ability to track motor symptoms in at-home settings have reported high compliance and clinical utility^26,53–57^.

In 2011, Chen et al. introduced *MercuryLive*, a web-based system that integrated data from wearable sensors and qualitative patient surveys for real-time, in-home monitoring of symptoms. Specifically, the system was used to guide potential changes in medications for patients with later-stage disease^58^. The advantage of such systems over sensor-only platforms is the ability to more seamlessly collect qualitative patient data, allowing clinicians and researchers to better contextualize quantitative sensor data. Other web application-based systems, like the *PERFORM* system presented by Cancela et al. in 2013, continued deploying wearable accelerometers and gyroscopes, but expanded the functionalities of the associated web application to include medication adherence questionnaires, food diaries, and the PDQ-39 questionnaire^59^, further expanding the qualitative information that supplements the objective data collected by wearable devices.

In-home monitoring became even more practical following the adoption of smartphones and other smart devices^60,61^. In 2011, Kostikis showed the feasibility of remote tremor monitoring using an Apple iPhone 3 G’s built-in accelerometer and gyroscope^62^. As recently as 2020, van Brummelen et al. tested seven consumer product accelerometers in smartphones (e.g., iPhone 7) and consumer smart devices (e.g., Huawei watch) and found that these products performed comparably to laboratory-grade accelerometers when assessing the severity of certain PD symptoms^63^. Smart tablets have also been shown to be helpful through the use of spiral drawing tests whose results significantly correlated with UPDRS scores and with the results of other tests including the *BRAIN Test*^64^.

Further expansion of smart devices came with the advent of user-friendly mobile applications such as the *Fox Wearable Companion* app developed by the Michael J. Fox Foundation. Silva de Lima et al. showed that using the app along with an *Android* smartphone and *Pebble* smartwatch resulted in high patient engagement and robust quantitative and qualitative data collection for clinicians to monitor PD progression and medication adherence^65^. Prince et al. report success using an independently designed iOS application^66^. Use of smartwatches in conjunction with such mobile applications also allows for cloud-based data storage, thereby enabling research and clinical teams to more effectively monitor symptom progression and severity in real-time^67^. In 2021, Powers et al. developed the “Motor fluctuations Monitor for Parkinson’s Disease” (MM4PD) system that used continuous monitoring from an Apple Watch to quantify resting tremor and dyskinesia. MM4PD strongly correlated with evaluations of tremor severity, aligned with expert ratings of dyskinesia, and matched clinician expectations of patients 94% of the time^2^. Multiple other groups, including Keijsers et al., have presented solutions that can assess motor fluctuations in real or simulated home settings using either wearable sensors^68–71^ or smart devices^72,73^. These types of solutions are particularly important for PD monitoring since assessing symptom fluctuations can give clinicians insight into medication dosing, disease severity, and even symptom triggers (e.g., a patient has worse tremor when driving compared to washing dishes). Monitoring fluctuations using smart devices can be particularly useful, as the device can document what a patient was doing when symptoms worsened, what time of day it happened, among other important environmental factors, providing clinicians a more wholistic picture of a patient’s disease. Data collected from fluctuation monitoring could also inform whether certain patients might be candidates for procedures such as deep brain stimulation.

Finally, multiple studies have proposed using technologies other than accelerometers and gyroscopes (either stand-alone or in smartphones). Instead, some studies used computer vision-based algorithms to assess data from video cameras, time-of-flight sensors, and other motion devices^74–76^. In the future, similar video analysis technologies could be combined with existing video platforms (e.g., *Zoom, FaceTime*) to regularly and reliably monitor motor impairments outside of the clinic. Significant work has also been conducted assessing the feasibility of using voice recordings to monitor and even diagnose PD. Arora et al. analyzed at-home voice recordings and were able to determine patients’ UPDRS scores to differentiate between patients with PD and healthy controls with a sensitivity of 96% and specificity of 97%^77^. Similar work on voice data from smartphones has been reported by many others^78–80^, indicating that voice analyses might be beneficial when developing technologies for monitoring and diagnosing PD.

### Computational approaches

Non-ML techniques to evaluate PD symptoms have evolved considerably over the last 30 years (Fig. 1B). Prior to adoption of machine learning algorithms, researchers used more traditional statistical and frequency domain analysis techniques. This likely occurred for two main reasons: (1) requisite computing power for ML was not as widely available and (2) the datasets collected in early studies were relatively less complex with respect to size and noise. Additionally, certain key machine learning techniques (e.g., backpropagation applied to neural networks) were not popularized until the late 1980s and early 1990s, with more widespread adoption occurring many years after with the advent of machine learning software libraries^81,82^. One of the first studies was in 1973 where Albers et al. showed that Parkinsonian hand tremor power spectra were easily distinguished that of control patients^83^ (Table 3). Statistical testing of frequency power spectrum also showed a significant correlation between selected features such as the total power of the frequency power spectrum and clinical ratings for dyskinesia severity^84^. Edwards et al. showed that combining multiple tremor characteristics (e.g., amplitude, dominant frequency) into one single index could also differentiate PD from non-PD movement^85^. Further development of computational techniques included applying more advanced regression models to data collected through different modalities (e.g., accelerometers, mechanical devices)^86,87^.

**Table 3:** Progression of non-machine learning techniques used to monitor and assess PD symptoms.

Authors, Year	Primary symptom(s)	Device(s)	Selected features	Key takeaways
Albers et al.^83^	Tremor	Hand-controlled force-measuring stick	Frequency and power spectrum	Parkinsonian tremor-power spectra are easily distinguished from control power spectra, providing an additional descriptive measure of tremor.
Blin et al.^88^	Gait	Potentiometer	Spatiotemporal and kinematic features	Relationships between certain spatiotemporal parameters were preserved (e.g., linear relationship between velocity and stride length) in PD and control.
Burkhard et al.^84^	Dyskinesia	Gyroscope	RMS of the frequency power spectrum between 0–20 Hz	Frequency power spectrum (FPS) values showed a statistically significant correlation with the clinical ratings for dyskinesia severity.
Edwards et al.^85^	Tremor	Laser-based hand displacement tracker	Tremor amplitude, frequency, and power spectrum	Multiple characteristics of tremor (e.g., amplitude, frequency) can be combined to form an index that can differentiate PD from non-PD.
Lewis et al.^90^	Gait	Force-sensitive resistors, video cameras, force plates, and EMG	Velocity, cadence, and stride length	Compared to age-matched control subjects, PD patients demonstrated 24% reduction in gait velocity and 23% reduction in stride length.
Matsumoto et al.^86^	Tremor	Accelerometer	Accelerations	Autoregression model parameters and the main tremor frequency can be used to differentiate between PD and non-PD (control and essential tremor)
Salarian et al.^155^	Gait	Tri-axial accelerometers and gyroscopes	Kinematic walking data	PD patients had significantly different gait compared to controls (e.g., 52% lower stride velocity). Patients with deep brain stimulation devices had significant gait improvement.
Sofuwa et al.^91^	Gait	Force plates, video cameras	Kinematic walking data	PD patients showed significant reduction in step length, walking velocity, and ankle plantarflexion compared to controls.
Elble et al.^87^	Tremor	Graphics tablet, accelerometer, and mechanical-linkage device	Tremor characteristics (time and frequency domain)	Analyses of 5 different tremor datasets revealed a logarithmic relationship between a 5-point tremor rating scale and tremor amplitude.
Chien et al.^156^	Gait	*GAITre* pressure-sensitive walkway	Kinematic walking data	Stride length is the best indicator of UPDRS III score improvements
Moore et al.^157^	Gait	Uniaxial accelerometer	Vertical linear acceleration and pitch angular velocity of left leg	Power analysis revealed distinctions between FoG and standing (e.g., high-frequency leg movements, overall power) which resulted in FoG detection accuracy of 89%.
Giuffrida et al.^158^	Tremor	*Kinesia* sensor (tri-axial accelerometer and gyroscope)	Tremor characteristics (time and frequency domain)	Tremor characteristics determined from the sensor correlated with clinician scores.
Hwang et al. (2009)	Tremor	Displacement-transducing laser	Tremor characteristics (time and frequency domain)	PD patients demonstrated abnormal tremor modulation compared to healthy controls in a load-bearing task.
Kim et al.^89^,	Bradykinesia	Gyroscope	Peak and total power in power spectrum of angular velocity	All features showed significant differences between control and PD patients and significant correlations with clinical finger tap score.
Sant’Anna et al.^159^	Gait	Inertial measurement unit and gyroscopes (uni- and bi-axial)	Kinematic walking data	A novel “symmetry index” of upper and lower limb movement during gait had an AUC of ~0.87 in differentiating between PD and control.
Palmerini et al.^160^	Gait	Smartphone	Kinematic walking data	10 principal components from PCA accounted for more than 90% of the variance in the original data. The first principal component accounted for 33% of the variance and was most correlated with standard deviation of step duration.
Stamatakis et al.^161^	Bradykinesia	Tri-axial accelerometer	Finger-tapping characteristics (e.g., movemet frequency, acceleration, number of halts, number of hesitations)	Logistic regression model trained using selected features achieved an AUC of 0.92 in classifying patients based on MDS-UPDRS scores.
Cancela et al.^59^	Gait	Tri-axial accelerometers	Step frequency, stride length, stride speed, arm swing, and entropy	Step frequency, stride length, arm swing, and entropy vary significantly between the OFF and ON state. Arm swing and entropy vary the most.
Buchman et al.^162^	Gait	Tri-axial accelerometer and gyroscope	Kinematic walking data	Regression revealed that performance on different mobility tasks (e.g., walking, sit to stand) was associated with Parkinsonian gait. Different tasks accounted for a wide range of gait variance.
Nair et al.^92^	Gait	Tri-axial accelerometer	Kinematic walking data	Logistic regression had accuracy of 94%, specificity of 96%, and sensitivity of 89%

Many studies also found success through standard hypothesis statistical testing such as t-tests and ANOVAs. Blin et al. used an in-lab potentiometer-linked string and pulley system to collect data on stride length. Using a Mann-Whiteney U test and linear regression, they found that variability of stride length was significantly more marked in PD patients and increased with Hoehn and Yahr clinical stages^88^. ANOVA conducted on finger tapping data (e.g., RMS angular velocity, RMS angular displacement) showed significant differences between PD and control subjects^89^.

To harness insights about gait abnormalities, researchers incorporated kinematic analyses into their studies. Using ANOVA on kinematic measurements of gait, Lewis et al. found that patients with Parkinson’s displayed lower gait velocity and stride length, but comparable cadence relative to healthy controls while exhibiting reductions in peak joint angles in the sagittal plane and reductions in ankle plantarflexion at toe-off of the gait cycle^90^. These gait and kinematic characteristics were corroborated using spatiotemporal analysis conducted by Sofuwa et al., who showed that patients with PD had a significant reduction and step length and walking velocity compared to control, with the major feature defining the PD group being a reduction in ankle plantarflexion^91^. More recently, Nair et al. used standard logistic regression on centroids from k-means clustering of data from tri-axial accelerometers to classify PD and control subjects with an accuracy of ~95%, specificity of ~96%, and sensitivity of ~89%^92^.

In more recent literature, machine learning techniques have proven to be highly effective in identifying PD symptom characteristics, especially when applied to varied datasets obtained using smart devices (Fig. 1B). The literature demonstrates strong performance across multiple machine learning techniques. Both neural network and non-neural network algorithms achieved high sensitivities and specificities in classification of PD symptoms using both raw and processed data. (Table 4) .

**Table 4:** Progression of machine learning techniques used to monitor and assess PD symptoms.

Authors, Year	Primary symptom(s)	Device(s)	Selected features	Supervised or unsupervised	Key takeaways
Jakubowski et al.^123^	Tremor	Accelerometer	30 statistical features	Supervised	Using “higher order” statistical characteristics of tremor (e.g., spectral moments of polyspectra) lead to average error < 3% in distinguishing between PD, essential, and physiological tremor
Keijsers et al.^163^	Hypokinesia, bradykinesia, and tremor	Six tri-axial accelerometers	Kinematic features (e.g., mean gait velocity, tremor frequency)	Supervised	A neural network with 2 hidden units and 4 input parameters resulted in a sensitivity and specificity of 100 and 98%, respectively.
Roy et al.^124^	Tremor	Accelerometer and EMG	Spectrum data	Supervised	Combining accelerometer and EMG data led to a global error rate < 10%.
Kostikis et al.^101^	Tremor	iPhone	Tri-axial accelerometer data	Supervised	Bagged decision trees had the highest AUC (0.94).
Lee et al.^103^	Dyskinesia	Accelerometer	Correlation between accelerometers on and entropy of accelerometer time series	Supervised	The proposed method (data pre-processing + random forest) significantly improves the estimation of limb-specific clinical scores.
Martinez-Manzanera et al.^107^	Bradykinesia	Accelerometer, gyroscope, and magnetometer	Raw and smooth splined signals	Supervised	Lowest classification error was ~33% for finger tapping, ~33% of diadochokinesis, and ~30% for toe tapping.
Memedi et al.^125^	Dyskinesia	Hand-held touch screen device	Characteristics of spiral drawing task	Supervised	Multilayer Perceptron classified the motor symptom (bradykinesia or dyskinesia) with an accuracy of 84% and ROC AUC 0.86 in relation to classifications of raters.
Rigas et al.^99^	Tremor	Microsoft *Band* on wrist	Accelerometer and gyroscope readings	Supervised	Decision trees achieved accuracy of 94% with 0.01% false positives.
Alam et al.^164^	Tremor	Tri-axial accelerometer and gyroscope	Tremor characteristics (time and frequency domain)	Supervised	Linear kernel SVM trained on data collected from the index finger classified rest and postural tremor severity with ~89% and ~82% accuracy, respectively.
Jeon et al.^113^	Tremor	Accelerometer and gyroscope	Temporal and frequency characteristics of tremor	Supervised	The best performing machine learning method changed depending on the type of tremor being assessed (e.g., resting tremor: Polynomial SVM, resting tremor with mental stress: Decision tree)
Butt et al.^126^	Tremor	Inertial measurement units	Movement characteristics (e.g., velocity, frequency) of multiple tasks (e.g., thumb–forefinger tapping, hand opening/closing)	Supervised	Neural network (accuracy of ~83%) outperformed SVM and logistic regression in distinguishing between slight-mild and moderate-severe PD
Oung et al.^127^	Tremor	Accelerometer, gyroscope, and magnetometer	Tri-axial accelerometer data	Supervised	Maximum average accuracy of ~91% using ELM, ~90% using PNN, and ~87% using KNN.
Jeon et al.^113^	Tremor	Tri-axial accelerometer and gyroscope	Tremor characteristics (time and frequency domain)	Supervised	Decision tree trained with features selected using PCA performed the best in predcting UPDRS scores. However, multiple ML algorithms performed well as measured by accuracy and RMSE.
Samà et al.^165^	Bradykinesia	Tri-axial accelerometer	Gait characteristics (time and frequency domain)	Supervised	SVM detected bradykinesia with ~91% accuracy. Regression models estimated UPDRS scores ~10% error.
Gao et al.^105^	Gait	Multiple across 2 separate studies	Numerous, including: demographic, gait, balance, and imaging data	Supervised	Feature selection streamlined large datasets. Model-free ML techniques (e.g., Neural Networks, SVM) performed better than model-based techniques (e.g., regression) in forecasting falls.
Bazgir et al.^115^	Tremor	Android smartphone with tri-axial accelerometer and gyroscope	Mean and max power spectrum density	Supervised	Naïve Bayes achieved 100% accuracy in classifying UPDRS scores of PD patients. NN had accuracy of ~93%, KNN of ~87%, and SVM of ~75%.
Butt et al.^116^	Tremor	Infrared-based motion detector	Speed and frequency of movement tasks	Supervised	Naïve Bayes on features selected by SVM performed the best.
Zhan et al.^72^	Gait	Smartphone	Features from gait, finger tapping, and voice tests	Unsupervised	mPDS correlated strongly with Hoehn and Yahr stage (0.91), MDS-UPDRS part III (r = 0.88), and MDS-UPDRS total (0.81).
Kim et al.^129^	Tremor	Accelerometer and gyroscope	Tri-axial accelerometer and gyroscope readings	Supervised	3-layer CNN resulted in ~85% accuracy when estimating UPDRS scores.
Pereira et al.^130^	Tremor	Smartpen with accelerometer	Time series images of 4 drawing tasks and 2 wrist movements	Supervised	CNNs performed best when utilizing data from each task separately and when combining data from each task
Vivar et al.^102^	Tremor	Infrared-based hand/finger motion detector (*Leap Motion Controller)*	9 texture features (e.g., mean, variance, energy, correlation)	Supervised	Bagged tree classifier using contrast achieved a 98% accuracy in classifying patients’ UPDRS scores.
Hssayeni et al.^104^	Tremor	Motion sensor	X, Y, and Z axis signal power	Supervised	Gradient tree boosting outperformed LSTM when estimating UPDRS-III scores.
Rehman et al.^106^	Gait	Tri-axial accelerometer and instrumented force mat	Gait kinematic features	Supervised	SVM performed better than random forest. Classification of PD versus control was significantly more accurate when using accelerometer data compared to using force mat data.
Rehman et al.^106^	Gait	Instrumented force mat	Gait kinematic features	Supervised	Random forest achieved the highest classification accuracy of 97% with 100% sensitivity and 94% specificity.
Rios-Urrego et al.^114^	Tremor	Tablet with stylus	Kinematic and spatial features from drawing task	Supervised	KNN using kinematic features and the kinematic + spatial + NLD features was most accurate (83%).
Steinmetzer et al.^166^	Gait	Tri-axial accelerometer and gyroscope	3D Euler angle and linear acceleration of the arms	Supervised	A three layer CNN detected motor dysfunction with an accuracy of 93% based on data from a timed up and go test.
Aich et al.^71^	Gait	Accelerometer	Gait kinematic features	Supervised	Decision tree had the highest accuracy of 88%, sensitivity of 93%, and specificity of 91%
Aich et al.^71^	Gait	Tri-axial accelerometers	Gait kinematic features	Supervised	Random forest had an accuracy of 96%, SVM of 93%, Naïve Bayes of 88%, and KNN of 86% classifying between the medication ON and OFF states.
Reches et al.^32^	Gait	*Opal* sensors (accelerometer, gyroscope, and magnetometer)	Time and frequency domain features	Supervised	SVM had ~80% sensitivity, ~83 specificity, and ~87% accuracy.
de Araújo et al.^112^	Tremor	Triple-axis gyroscope and an accelerometer	Time and frequency domain tremor features	Supervised	KNN outperformed 6 other machine learning algorithms in classifying hand resting tremor in patients with PD.
Sajal et al.^80^	Tremor	Smartphone-based accelerometer	Time and frequency domain features	Supervised	KNN had the highest accuracy for both PD vs non-PD and UPDRS 0–4 classifications
Moon et al.^119^	Gait	6 IMU sensors	Gait and postural sway features	Supervised	The accuracy of the models in classifying between PD and ET ranged from 0.65 (KNN) to 0.89 (NN).
Veeraragavan et al.^122^	Gait	8 force sensors on the soles of each foot	Swing, stance, and force metrics	Supervised	The neural network used for PD diagnosis had an accuracy of 97%. H&Y staging was conducted with an accuracy of 87%.
Shi et al.^128^	Gait	3 IMU sensors with tri-axial sensors	Wavelet transformed gait data	Supervised	CNN achieved an accuracy of 89% using wavelet-transformed data, 84% using FFT of the time series, and 74% using raw time series
Sigcha et al.^131^	Gait	IMU sensor	Time and frequency domain features	Supervised	Highest AUC (0.94) was achieved with CNN-LSTM using FFT + 3 previous windows when classifying presence vs non-presence of FOG.
Ibrahim et al.^132^	Tremor	Motion sensor	1D time-domain tremor amplitude signal	Supervised	CNN with perceptron was used to estimate amplitude of future tremor at time steps of 10, 20, 50, and 100 ms. Accuracy ranged from 90–97%.
Channa et al.^108^	Bradykinesia	Accelerometer and gyroscope	Time and frequency domain features	Supervised	Bradykinesia classification had a sensitivity of 100% and specificity of 89%
Mirelman et al.^110^	Gait	Tri-axial accelerometers and gyroscopes	Gait kinematic features	Supervised	Different gait features held varying levels of importance for distinguishing between stages of PD (AUC of 0.76–0.9). Upper-limb features best discriminated controls from early PD, turning features were important in mid-stage PD, and stride-related features were more important in more advanced stages.
Rupprechter et al.^111^	Gait	KELVIN-PD video recording platform	Gait kinematic features	Supervised	Random forest highly aligned with clinical examiners’ ratings of UPDRS scores (rarely differed by more than one point; 95% agreement) when trained on computer vision-derived features.
Liu et al.^167^	Tremor	Tri-axial accelerometer	Tremor characteristics (time and frequency domain)	Supervised	SVM achieved the best performance with an overall accuracy of ~95% when differentiating between patients with different tremor severities

The vast majority of machine learning techniques are supervised.

There is still significant research being conducted on optimizing and refining most of the ML algorithms discussed here, as many aspects of ML design still work through trial and error. This applies to both determining model parameters (e.g., learning rates for gradient descent, impurity levels in decision trees) and selecting algorithms themselves (e.g., neural network versus decision tree)^93–96^. In reality, multiple different models could be effective in performing the same task on a given set of data^97,98^. Here, we present objective measures of ML model performance while also attempting to provide rationale regarding the design criteria that may have led researchers to choose one algorithm over another.

Non-neural network machine learning algorithms have proven effective in Parkinson’s disease classification, as they often provide more mechanistic insight/interpretability and generally require less training data compared to neural networks. Multiple studies have found that decision trees are highly effective in classifying Parkinson’s versus control patients based on accelerometer and gyroscope data. Using data from a Microsoft *Band* smartwatch, Rigas et al. used decision trees to achieve a tremor detection accuracy of 94% with a 0.01% false positive rate^99^. Aich et al. showed that a decision tree trained on gait characteristics such as step time and length, stride time and length, and walking speed distinguished Parkinson’s patients from healthy controls with an accuracy of ~88%, sensitivity of ~93%, and specificity of ~91%, outperforming k nearest neighbor (KNN), support vector machine (SVM), and Naïve-Bayes^100^. The design choices in these studies were conducive to using decision trees, as there were multiple quantitative variables (e.g., stride length) with specific cut-offs (e.g., stride length <1.2 m) that informed certain diagnoses. Decision trees also enabled researchers to quantitatively determine which feature(s) (e.g., tremor frequency) from the data were most important in determining final classifications, thus improving the link between data analysis and understanding of disease.

While decision trees can be effective, they can also overfit training data, thereby limiting their generalizability. Therefore, many groups have found success using bagged decision trees, a technique that trains multiple trees using subsets of the training data and then aggregates the final results. Bagged decision trees can be particularly useful to mitigate overfitting that can result from analyzing relatively small datasets. Kostikis et al. used data from 25 patients with PD and 20 health controls and found that bagged decision trees on tremor features resulted in an AUC of 0.94, higher than any other algorithm they tested (e.g., logistic regression, SVM, AdaBoost)^101^. In a study with 20 patients with PD, bagged trees showed between 95 and 98% accuracy in classifying patients as per the MDS-UPDRS 0,1,2 scheme when using tremor data from motion sensors rather than accelerometers or gyroscopes^102^.

Results continued to be strong with a variant of bagged decision trees known as random forests (RF), which can be useful in improving accuracy and further reduce overfitting, with the tradeoff of longer training times. RF performed better than logistic regression on features from gait analysis, sway tests, and time up-and-go tasks when classifying between progressive supranuclear palsy and Parkinson’s and were also useful in estimating clinical scores of dyskinesia^103^. At the same time, researchers have encountered success with another variation of decision trees known as boosted trees, with gradient tree boosting outperforming a long short-term memory neural network when estimating UPDRS-III scores based on motion sensor data from the wrist and ankle^104^.

To further improve algorithm efficiency and reduce computational cost, researchers have leveraged feature selection techniques in combination with established machine learning algorithms. Feature selection is particularly important in the design of studies that evaluate multiple ML algorithms to identify the top performers or train algorithms on different datasets^105,106^. Feature selection is also commonly used as a tool to help improve algorithm performance. When used in conjunction with feature selection techniques such as recursive feature elimination, RF achieved a classification accuracy of 96% when grading gait abnormalities of PD patients on and off medications^71^. Another type of SVM-based feature selection was useful in achieving high RF performance when classifying PD vs non-PD patients, resulting in accuracy of 97%, sensitivity of 100%, and specificity of 94%. In general, many different feature selection techniques have shown to be useful with multiple ML algorithms^32,106–108^. SVMs have shown to perform well with and without feature selection before model training^32,106^.

Feature analysis, however, does not stop with feature selection. Specifically, post-hoc feature importance calculations can be beneficial in better understanding why specific models work the way they do, providing more insight related to the clinical applications of the model. Rehman et al. built multiple partial least discriminant analysis models using subsets of gait features measured in patients with PD and healthy controls, and used feature importance metrics to identify that, among others, step velocity, step length, and gait regularity were the most influential features in the model. This type of analysis is particularly beneficial, as they can improve clinical decision-making independent of using machine learning models, by providing clinicians with more nuanced signs/symptoms of early disease manifestation or disease progression^109^. Similar analyses were conducted on gait abnormalities by Mirelman et al., who stratified patients based on their PD disease progression and found that different features were more important in differentiating between various stages of PD^110^. For example, as PD progressed, features related to more challenging activities such as turning became more important for patient classification, but Mirelman et al. found that this increase in importance occurred in earlier stages of disease than one would normally expect. Similar analyses were reported by additional groups investigating gait and even other symptoms of PD^104,111,112^.

While the choice of which ML algorithm to use can partially be informed by the type of data, size of the study, etc., some papers have shown that the accuracy of a machine learning model depends on the type of tremor being evaluated, further highlighting the inherent trial-and-error nature of ML study design. Jeon et al. found that while decision trees were most accurate when classifying patients based on resting tremor with mental stress and intention tremor, resting tremor classification alone was most accurate with polynomial SVM and postural tremor classification was most accurate with (KNN)^113^. In the same vein, multiple groups have found that KNNs using time and frequency domain data are highly effective in Parkinson’s versus control classification^80,112,114^ using tremor data. Finally, Butt et al. and Bazgir et al. in 2018 both found that Naïve Bayes outperformed other tested algorithms when classifying Parkinson’s tremor using motion and accelerometer/gyroscope data, respectively^115,116^.

A few unsupervised learning algorithms have been developed for PD classification. Unsupervised learning can be useful when designing studies with large datasets that might be too cumbersome to manually label—a pre-requisite for training supervised ML models. Unsupervised learning is also beneficial in exploratory analyses to provide structure and novel insights from large and diverse datasets. Zhan et al. developed a novel “Disease Severity Score Learning” algorithm that calculated a “mobile Parkinson disease score” (mPDS) based on 435 features from gait, finger tapping, and voice tests that were conducted using smartphones. mPDS scores strongly correlated with MDS-UPDRS part III, MDS-UPDRS total, and Hoen and Yahr stages. This work represents ongoing efforts to create more objective measurements of Parkinson’s disease progression that are not impacted by interrater variability^72^.

The development of artificial neural networks to study large datasets have recently been used for PD symptom classification. Neural networks have multiple use cases but are most often utilized on large sets of data whose features must be combined using complex, non-linear relationships for classification or regression tasks. That being the case, neural networks typically require more data to train compared to other ML algorithms and, as a consequence, are more computationally expensive. Though neural networks can be powerful tools, they tend to be more “black box”, lacking in interpretability compared to other ML algorithms^52,117,118^. Even so, neural networks are one of the most popular ML algorithms used today and have achieved strong performance when applied to diagnosing and monitoring PD.

Moon et al. used 48 features across gait and postural sway collected from six inertial measurement units (IMUs) across patients’ backs, upper extremities, and lower extremities to differentiate between PD and essential tremor. After testing multiple machine learning algorithms (e.g., SVM, KNN, neural network, logistic regression), the authors found that a neural network with a learning rate of 0.001 had the highest accuracy (0.89), precision (0.61), and F1-score (0.61)^119^. Moon et al.’s paper is a good example of the design process often times used with machine learning in that multiple algorithms are tested before selecting one algorithm with specific hyperparameters (e.g., learning rate, number of hidden layers) that are also typically selected with trial and error^120,121^. Veeraragavan et al. also used neural networks, but attempted two different tasks: classifying between PD and healthy patients based on gait and classifying PD patients into Hoehn and Yahr clinical stages. Parkinson’s versus healthy control classification was achieved with an accuracy of 97% using a single hidden layer network with 25 nodes, while classification into Hoehn and Yahr stages was accomplished with an accuracy of 87% using a single hidden layer network with 13 nodes^122^. These results suggest that neural networks are promising candidates for disease classification and staging.

Early efforts to apply machine learning to PD tremor data utilized single hidden layer perceptron classifiers of 30 higher order statistical characteristics of tremor accelerometer data as inputs to differentiate between Parkinsonian, essential, and physiological tremor^123^. Such efforts essentially combined sophisticated feature extraction with relatively simple algorithm architecture for classification tasks. Other approaches, such as the dynamic neural network used by Roy et al., aimed to classify tremor as “mild”, “moderate”, or “severe” (based on UPDRS), using spectrum data from EMG and accelerometer measurements. Leveraging input features that required minimal pre-processing, such as accelerometer signal energy after lowpass filtering, Roy et al. achieved global classification error rates of less than 10%^124^. Others have reported success using neural networks trained on similar features that require little pre-processing^125,126^. Alterations to classical neural networks have also performed well. Oung et al. showed that extreme learning machines—neural networks that learn weights without backpropagation—achieved 91% classification accuracy when tremor and voice data were used as inputs to the network^127^.

Convolutional neural networks (CNNs) have recently played a large role in Parkinson’s disease classification due to their ability to directly analyze image data. In many cases, this reduces the amount of feature extraction needed. For example, if using a CNN to analyze tremor data collected by accelerometers, researchers do not need to extract features such as frequency, amplitude, etc., because the input to the CNN can simply be a processed version of the accelerometry graph itself. In 2020, Shi et al. used graphs of wavelet-transformed data (decomposing the data into a set of discrete oscillations called wavelets) from tri-axial accelerometers, gyroscopes, and magnetometers as inputs to a CNN to classify FoG and non-FoG episodes. Overall, the CNN displayed classification accuracy of ~89%, sensitivity of ~82%, and specificity of ~96%. The same study found that CNNs using raw time series data or Fourier-transformed data as inputs did not perform as well^128^. This shows that researchers must carefully select pre-processing techniques when using CNNs, as this choice can significantly alter the algorithm’s performance. However, using Fourier-transformed data improved CNN-based tremor classification. Kim et al., in 2018, reported ~85% accuracy when estimating UPDRS scores using a 3-layer CNN with a soft-max classification final layer. Rather than extracting specific features from accelerometer data to use as inputs to the CNN, Kim et al. used a stacked 2D FFT image of the tri-axial accelerometer and gyroscope data^129^.

Researchers have experimented with various CNN architectures and structures as well. Pereira et al. compared CNNs with ImageNet or Cifar10 architectures to an optimum-path forest, support vector machine with radial basis function, and Näive-Bayes using data from 4 drawing (e.g., spiral drawing) and 2 wrist movement tasks to distinguish Parkinson’s from control patients based on tremor. Overall, the CNNs outperformed the other machine learning techniques with respect to classification accuracy when using data from each aforementioned task separately (single-assessment case) and when combining data from each task (combined-assessment case)^130^. Sigcha et al. in 2020 wanted to model the time-dependencies of FoG and used a novel CNN structure by combining a classical CNN with a long short-term memory (LSTM) recurrent neural network to classify FoG and non-FoG episodes. Using Fourier-transformed data from an IMU on patients’ waists as an input, the CNN-LSTM combination achieved an AUC of 0.939^131^.

CNNs have also been useful beyond classification tasks. In 2020, Ibrahim et al. used a CNN with perceptron to estimate the amplitude of future tremor at 10, 20, 50, and 100 millisecond time steps, with a prediction accuracy ranging from 90 to 97%^132^. Both traditional and convolutional neural networks will likely continue to be useful in machine learning-based analysis of PD symptoms.

### Interplay between technology and computational techniques

The technology and computational techniques used to monitor PD motor symptoms have evolved concurrently. As technology improves, different computational techniques must be developed and optimized to handle increasing amounts of data collected by new devices. The same applies in reverse. As advancements are made in computation that enable researchers to ask and answer different questions, new technologies must be developed that can facilitate these new analyses.

The overarching, major change seen in the technology used to diagnose and monitor PD over the last ~50 years has been the transition from laboratory to home monitoring. This technological evolution has undoubtedly been accompanied by a shift in computational approaches. Fundamentally, the techniques used to analyze data collected in well-controlled laboratory settings must be different from those required to analyze data collected in real-world conditions. As such, the evolution of technology necessitated computational methods that could: (1) better denoise signals, (2) make predictions given large sets of structured data, and (3) make predictions given large sets of unstructured data.

In-lab diagnosis and monitoring of PD generates data with less noise compared to data generated from real-world monitoring. This manifests in two ways. First, the data signal itself contains less ambient noise. For example, by using high-quality microphones or working in sound-treated rooms, researchers can control for room noise if recording voice samples from patients with PD^133,134^. At another level, the data from most in-lab studies are “de-noised”/simplified due to the inherent structure built into these studies. Assessing gait abnormalities via the timed up-and-go test or quantifying tremor via circle drawing tests produces highly consistent and uniform data since participants have executed the same task(s) in the same way to generate the data. This is not the case in real-world settings. As technology enabled real-world data collection, de-noising became one of the first priorities, both through simple filtering^77^ and data labeling (e.g., smartwatch labeling if a participant was running, swimming, sleeping)^135^. Though, apart from adding functionalities to deal with noisy data, foundational computational techniques such as frequency analyses and statistical testing were still adequate.

The adoption of machine learning generally correlated with the ability to collect increasing amounts of data, which have enabled researchers to ask new questions. The prime example of this is the adoption of smart devices. Before, researchers could ask participants to wear accelerometers, gyroscopes, heart rate monitors, etc. to collect varied types of data. Smart devices enabled device consolidation, improving ease of use for patients, and therefore increasing the amount of data that could be collected. Even more, smart devices improved ease of collecting qualitative data. Instead of relying on patient diaries or recall from memory, app-based monitoring on phones or tablets allowed patients to more seamlessly provide qualitative data related to medication adherence, exercise levels, mood, etc.

With access to increased volumes and types of data, researchers and clinicians started asking questions that were more suited for analysis with ML rather than non-ML techniques. These questions can broadly be assigned into two categories: (1) predictions and (2) classifications. When investigating PD, researchers were interested in predicting severity of symptoms and disease progression, while classifying patients for diagnostic and therapeutic purposes. ML algorithms were specifically suited for this task given their ability to leverage non-linearities and more efficiently handle large datasets. For example, neural networks enabled researchers to uncover complex, non-linear relationships between quantitative (e.g., tremor frequency) and qualitative (e.g., medication adherence) data to predict UPDRS scores, while SVM allowed for high-dimensional (>3 independent variable) classification. With smart devices providing access to vast amounts of data, researchers leveraged algorithms such as random forest that parallelized classification and prediction tasks, making data analyses more efficient and insightful.

It is clear that the computational techniques and technology used to monitor PD have co-evolved over the years. As technology advances, new computational techniques will be required to take advantage of the technologies’ improved functionalities and vice versa.

## Discussion

The technology used to monitor and quantify Parkinson’s motor symptoms has undergone a rapid transformation in the past few decades. Early monitoring began with in-lab devices such as needle-based EMG, transitioned to using in-lab accelerometers/gyroscopes, then to more wearable accelerometers/gyroscopes, and finally to phone and mobile & web application-based monitoring in patients’ homes. The shift from in-lab to in-home monitoring will enable physicians to make more data-driven decisions regarding patient management. Along the same lines, significant progress has been made with respect to the use of machine learning to classify and monitor Parkinson’s patients. Using data from multiple different sources (e.g., wearable motion sensors, phone-based accelerometers, video cameras), researchers have designed both neural network and non-neural network-based machine learning algorithms to classify/categorize Parkinson’s patients across tremor, gait, bradykinesia, and dyskinesia. Further advancements in these algorithms will create more objective and quantitative ways for physicians to diagnose and manage patients with Parkinson’s.

As machine learning becomes more prevalent in medicine, regulators such as the Food and Drug Administration (FDA) are developing new protocols to assess the safety and efficacy of ML-based health technologies. The plan outlined by the FDA to improve evaluation of these technologies includes: (1) outlining “good machine learning practices”, (2) setting guidelines for algorithm transparency, (3) supporting research on algorithm evaluation and improvement, and (4) establishing guidelines on real-world data collection for initial approval and post-approval monitoring^136^. As this plan goes into action over the next few years, trial endpoints for diseases will still likely be established clinical metrics (e.g., UPDRS) rather than novel metrics generated by new ML-powered devices^137,138^. There seems to be, however, a future in which device-generated metrics replace or are used in conjunction with traditional clinical metrics. In the case of PD monitoring, the FDA’s approval of Great Lakes NeuroTechnologies’ *KinesiaU* device and provider portal to monitor motor symptoms of PD is a first step in that direction^139^. ML will undoubtedly play an increasingly larger role in medicine, and the FDA’s actions to navigate this new healthcare environment should be carefully monitored by researchers in this field.

Digital PD monitoring has enabled an understanding of patients’ symptoms to a level of detail not seen before. Prior to the adoption of wearable and smart devices in this field, clinicians were blind to the manifestation of PD motor symptoms outside of the clinic (e.g., brushing teeth, exercising, driving). Device-based monitoring has also helped fill in gaps left by sometimes inaccurate or incomplete patient diaries. However, many barriers exist to full clinical adoption of digital monitoring, including the cost of digital devices, lack of secure and reliable pipelines to transfer data to physicians, and perhaps the technological capabilities of patients with PD^140^. These barriers can start to be overcome through: (1) public-private partnerships that help lower the cost of digital devices for hospital systems to provide to their patients, (2) increased focus on data storage and retrieval infrastructure, and (3) patient education.

In the future, a transition to truly continuous PD symptom monitoring has the greatest potential by leveraging easy-to-use mobile applications on smart devices (e.g., smartphones, smartwatches) that can integrate quantitative and qualitative (e.g., quality of life surveys) data for physicians to better understand a patient’s experience with Parkinson’s. Further development of these applications, along with live data transmission and storage to the cloud will enhance the usability and utility of these technologies. Incorporating machine learning to these functionalities can then enable more objective disease staging/diagnoses by physicians and enhanced predictive capabilities for identifying disease progression. However, there is much work to be done related to developing better disease biomarkers to train these machine learning algorithms on. Reliable biomarkers must accurately identify symptoms of PD across patient populations and stages of disease. These biomarkers might also need to be different in different contexts (e.g., tremor during driving is different from tremor while brushing teeth). Identifying the nuances of digital biomarkers will be essential in realizing the full potential of machine learning and high technology in the monitoring of Parkinson’s symptoms.

## Methods

We queried the US National Library of Medicine PubMed database (PubMed). Five compound search terms were used to query PubMed for machine learning and computational publications and clinical trials: “Parkinson’s” + *SYMPTOM* + (1) machine learning, (2) neural network, (3) quantification, (4) analysis, and (5) monitoring where “*SYMPTOM*” was either “tremor”, “gait”, “bradykinesia”, or “dyskinesia”. These queries resulted in 10,200 papers. Manuscripts about technology for monitoring PD symptoms were identified in PubMed with advanced search terms: ((automatic detection) OR (classification) OR (wearables) OR (digital health) OR (sensors)) AND “Parkinson’s” + *SYMPTOM*. These queries resulted in 2600 papers. Studies were first de-duplicated and then excluded if they did not: have full text availability, use data from humans, or evaluate PD specifically. Book chapters, review articles, and “short communications” were also excluded. Titles and abstracts were reviewed before further assessing a sub-set of representative English language papers. These papers were selected as they best characterized the machine learning and technology timelines that manifested from reviewing the literature.

### Reporting summary

Further information on research design is available in the Nature Research Reporting Summary linked to this article.

## Supplementary information

Supplementary Information

Reporting Summary

## Data Availability

The data used to generate the figures and tables are publicly available to researchers through the National Library of Medicine. Additional inquiries are welcome to the corresponding author.
